# Obsessive-Compulsive Disorder (OCD): A Comprehensive Review of Diagnosis, Comorbidities, and Treatment Approaches

**DOI:** 10.7759/cureus.48960

**Published:** 2023-11-17

**Authors:** Abhimanyu Singh, Vaibhav P Anjankar, Bhagyesh Sapkale

**Affiliations:** 1 Medicine, Jawaharlal Nehru Medical College, Datta Meghe Institute of Higher Education and Research, Wardha, IND; 2 Anatomy, Jawaharlal Nehru Medical College, Datta Meghe Institute of Higher Education and Research, Wardha, IND

**Keywords:** treatment resistance, comorbidity, metacognitive, cortico-striatal circuitry, neuropsychiatric, exposure and response prevention (erp), serotonin reuptake inhibitors (sris), cognitive behavioral therapy (cbt), compulsions, obsessions

## Abstract

Obsessive-compulsive disorder (OCD) is a neuropsychiatric disorder widely recognized for its recurrent obsessions and compulsions, which may cause severe impairment worldwide. This review explores the difficulties in diagnosing OCD, its comorbidities, and its treatment approaches. Psychiatry and neuroscience face noteworthy obstacles in treating OCD, which is frequently misdiagnosed and inadequately addressed. This illness, which causes upsetting symptoms that interfere with day-to-day living, affects not only adults but also children and adolescents to a great extent. Despite the availability of multiple therapy methods, such as pharmacological and psychological approaches, many patients exhibit resistance, emphasizing the necessity for alternative therapies. OCD and other psychiatric conditions like bipolar disorder, schizophrenia, and attention deficit hyperactivity disorder substantially overlap, highlighting the complexity of mental health diagnoses. Furthermore, its comorbidity with these diseases further highlights OCD's intricacy. Several therapy considerations have been mentioned, such as using larger dosages of medications and combining different therapeutic approaches. Their association suggests possible common pathogenic pathways between OCD and other psychiatric illnesses. The review concludes that, given the significant number of people who still struggle with chronic symptoms, new treatment techniques and ongoing research are necessary, even in the face of improvements in the understanding and treatment of OCD.

## Introduction and background

Long-term recurrent thoughts (called obsessions), compulsive activities (called compulsions), or both can be symptoms of obsessive-compulsive disorder (OCD) [1]. The chronic and prevalent condition known as OCD is linked to significant disability worldwide. Obsessive-compulsive and related disorders are a group of conditions that are often underdiagnosed and undertreated. They are now included in the Diagnostic and Statistical Manual of Mental Disorders, Fifth Edition, and the International Classification of Diseases, Eleventh Revision. A severe and incapacitating psychiatric condition, OCD poses several difficulties for neuroscience. The cortico-striatal circuitry's neurological correlates and hypotheses about an imbalance between goal-directed and habitual behavior are analyzed and contrasted with metacognitive views [2]. The underlying processes of these treatments are currently being investigated. In children and adolescents, OCD is a debilitating illness characterized by a particular collection of upsetting symptoms, such as stressful, time-consuming rituals (compulsions) and persistent, intrusive thoughts (obsessions) [3]. Up to 2.5% of people will experience OCD at some point, resulting in significant morbidity [4]. The success of treatments for OCD varies, but they include psychological, pharmaceutical, and surgical methods [4]. A widespread, long-lasting, and frequently incapacitating disorder is OCD.

Serotonin reuptake inhibitor drugs and exposure and response prevention are the only proven first-line therapies for OCD. Still, a small percentage of individuals do not react to either technique, and even fewer achieve total remission [4]. Antipsychotic augmentation is the only pharmaceutical treatment for OCD with significant empirical support, aside from SRI monotherapy [5]. About 1% and 1.5% of adult males and females, respectively, suffer from obsessions or compulsions that result in social dysfunction or emotional misery [6]. Most adults with OCD have ongoing issues, whereas the other half experience episodes [6]. There were no appreciable variations in the intensity of the majority of anxiety symptoms between the groups of patients with generalized anxiety disorder (GAD) and OCD diagnoses [7]. Despite the enormous benefits reportedly associated with memantine augmentation, the routine use of memantine as an augmentation agent for OCD cannot be recommended yet [8]. The perception of the absurdity of obsessive thoughts and the knowledge that OCD symptoms never go away is rooted in incompleteness and depersonalization [9]. This narrative review article attempts to provide a thorough analysis of the current understanding of OCD, including its impact on various age groups, treatment approaches, and hereditary interactions. It also covers the condition's neurological foundations. The aim is to enhance comprehension of OCD by consolidating current knowledge, which could lead to improved diagnostic and treatment strategies.

## Review

Search methodology

**Figure 1:**
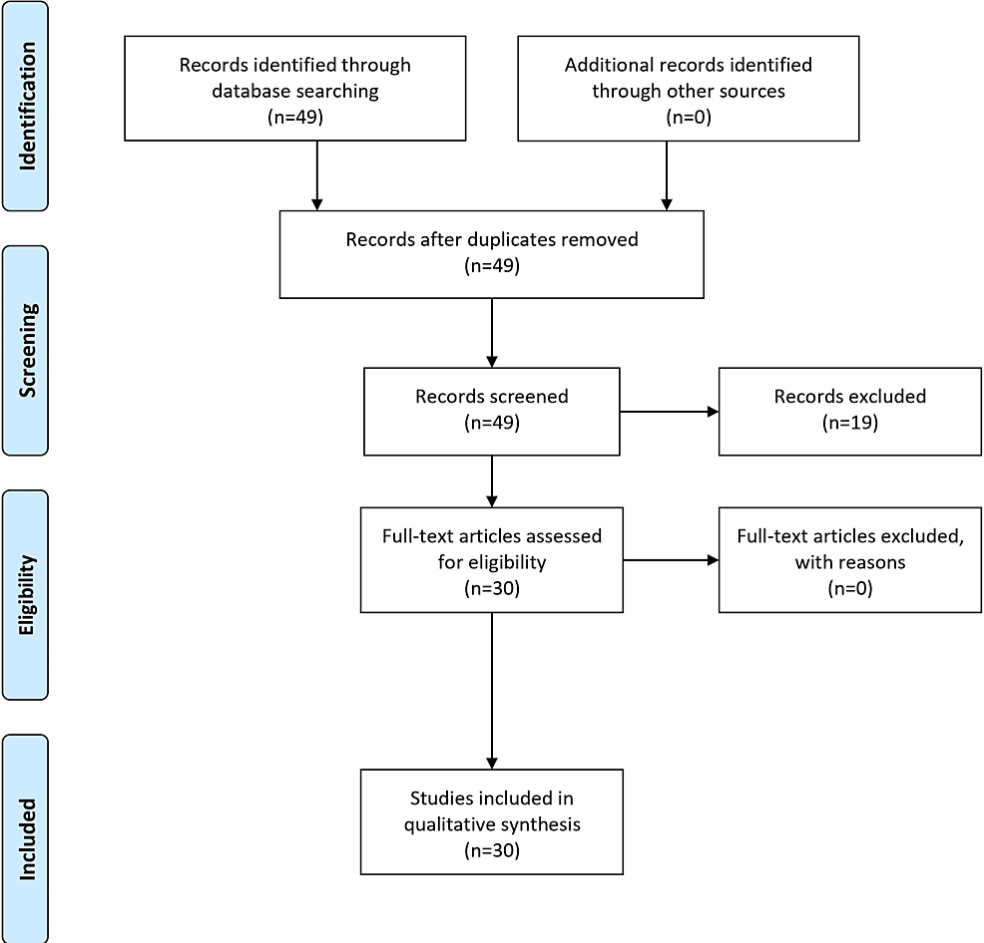
PRISMA flow diagram PRISMA: Preferred reporting items for systematic reviews and meta-analyses

A methodical approach using the major databases PubMed/MEDLINE, PsycINFO, Scopus, and Google Scholar was used in the search methodology for the comprehensive review of OCD. Psychiatric disorders, neuropsychiatric disorders, diagnosis, comorbidities, treatment methods, OCD, and obsessive-compulsive disorder (OCD) were among the terms included in the search technique, which combined medical subject headings (MeSH) phrases with keywords. Articles about OCD diagnosis, comorbidities, and treatment strategies were included in the inclusion criteria. These included research with adult and pediatric populations, investigations into neurological correlates, and talks about co-occurring mental disorders. The exclusion criteria ruled out articles with little or no information on the stated themes, research only on animal models, and studies not written in English. Initial screening of abstracts and titles was part of the research selection procedure. A full-text examination of papers that might be relevant was then conducted to determine eligibility based on eligibility and exclusion criteria. Next, for the chosen studies, data extraction was carried out. This review is primarily a narrative review that incorporates both systematic and narrative elements. Referencing and covering several facets of the condition, it methodically combines data on the diagnosis, comorbidities, and treatment modalities for OCD. Overall, the material is better understood because of the narrative components that add to a thorough overview and discussion of the difficulties of OCD. This dual approach strengthens the review's structure and ensures a thorough exploration of the chosen topics. The Preferred reporting items for systematic reviews and meta-analyses flow diagram is presented in Figure 1.

OCD is a chronic illness characterized by uncontrollably recurrent thoughts (obsessions), repetitive behaviors (compulsions), or both. Time-consuming symptoms are a common feature of OCD patients, and they can seriously disrupt everyday life or cause significant discomfort [10]. A widespread and frequently chronic mental condition that severely impairs a patient's functionality and quality of life is OCD. Excessive, inappropriate, and intrusive thoughts that evoke anxiety, along with time-consuming compulsions that significantly impair and disturb the sufferer, are the hallmarks of this condition [11]. The disease is not well understood by the general public or the professional community, and feelings of guilt and shame frequently accompany the symptoms, so evaluations that fail to consider these subtleties will frequently overlook OCD and mistakenly assign its symptoms to mood or anxiety disorders, for which essential treatment differences exist [11].

Furthermore, the intrusive thoughts associated with OCD may be confused with the delusions of postpartum psychosis. This can result in either failing to identify the latter as a psychiatric emergency or, more frequently, in misinterpreting and overreacting to OCD, leading to inappropriate actions that may involve engaging child services or a higher level of care than is necessary [12]. Recurring unwelcome ideas, pictures, or cravings are called obsessions (egodystonic). Repetitive behaviors or mental activities carried out strictly with regulations or to ease the anxiety associated with obsessions are known as compulsions [13]. Many psychiatric diseases can be aggravated or precipitated by childbirth; however, most of the work published thus far has been on mood disorders. Following childbirth, OCD may manifest by itself or in conjunction with other mental illnesses, such as major depressive disorder [14]. OCD may be misdiagnosed or underdiagnosed as a major depressive illness because patients and physicians are unaware of the connection between OCD and delivery.

Understanding the complexity of OCD

Panic disorder, social anxiety, agoraphobia, particular phobias, and GAD are among the anxiety disorders. Apart from the distinct indications of various illnesses, a shared sensation of anxiety and even dysphoria could exist among them. Naturally, therapy with the same or combination of medications is the norm [15]. Comparing individuals with GAD to those with OCD, the severity of phobic disorders, conversion disorders, cardiac autonomic dysfunctions, and hypochondria was significantly higher in the former group [15]. Anomalous, invasive sexual thoughts and pictures are a hallmark of OCD sexual obsessions. It is estimated that 16.8% of people with OCD experience sexual obsessions [16]. The WHO has listed OCD as one of the ten most incapacitating conditions [17]. The egodystonic aspect of OCD, which implies that those with it are fully aware of how irrational or excessive their conduct is, has been proposed as a possible contributing factor. The co-occurrence of other mental health issues with pediatric OCD increases the severity of the ailment and the difficulty in treating it [18]. Cognitive behavioral treatment (CBT) effectively treats OCD and depression and anxiety, two of its most prevalent comorbid illnesses [18]. As a result, treating OCD may also help symptoms of anxiety and depression in the long run. The three most common and debilitating psychiatric disorders are addiction, depression, and OCD, which have significant psychological and medical costs and higher morbidity and mortality rates [19]. A comprehensive overview of anxiety disorders and the multifaceted nature of OCD is shown in Table 1.

**Table 1:** A comprehensive overview of anxiety disorders and the multifaceted nature of OCD OCD: Obsessive-compulsive disorder

Category	Description
Types of anxiety disorders	Panic disorder, social anxiety, agoraphobia-specific phobias, generalized anxiety disorder
Commonalities among anxiety disorders	Shared sensations of anxiety and dysphoria, typically treated with similar medication or combinations
World Health Organization (WHO) ranking	OCD is one of the top 10 most incapacitating conditions
Egodystonic aspect of OCD	Patients are conscious of the irrational or excessive nature of their behavior
Co-occurrence with pediatric OCD	Other mental health issues increase the severity and difficulty of treatment.
Treatment	Cognitive behavioral treatment (CBT) is effective for OCD and its common comorbid illnesses, such as depression and anxiety
Most debilitating psychiatric disorders	Addiction depression OCD

OCD and schizophrenia: diagnosis and treatment

Deep brain stimulation (DBS) is becoming more and more popular as a treatment for addiction, OCD, and depression; nevertheless, administering DBS can be difficult because of the intricate interplay of neuronal circuits, the requirement for exact targeting, individual differences in brain anatomy, and potential adverse effects [19]. Compulsive disorder is a significant mental condition that is more prevalent than others. Treatment and diagnosis may be delayed due to the stigma and shame surrounding it and a failure to recognize its telltale signs [20]. Schizophrenia is one of the mental illnesses that can exhibit obsessive-compulsive-like symptoms. Descriptions of this date back to Kraepelin and Bleuler. This review highlights the concept of pseudo-obsession, which is frequently overlooked. The idea can enhance diagnostic procedures by helping to distinguish between genuine obsession in OCD and associated psychopathological symptoms in schizophrenia spectrum illnesses [21]. Effective psychological and pharmacological treatments are available for the distressing, time-consuming, repetitive thoughts and routines and the resulting functional impairment [3,20]. The range of responses to the pharmacological treatment of OCD depending on the type of symptoms, according to the analyses conducted, is not significant enough to support psychotherapy, as well as surgical treatments [22].

Parental modeling is when an OCD-afflicted parent models problematic behavior patterns daily, which can impact kids. Contrarily, family accommodation refers to the active involvement of parents in their child's obsessive rituals through the modification of daily schedules or the facilitation of avoiding OCD triggers to lessen the child's suffering and the amount of time spent carrying out compulsions [23]. Using medication would be another method of treatment. Regretfully, antipsychotic drugs rarely help with OCD symptoms. Antipsychotic drugs can be used to treat the symptoms of schizophrenia first. Treatment for obsessions would then begin when the psychotic symptoms have sufficiently resolved [23]. Though not proven, it is thought that some antipsychotic drugs, such as clozapine, cause people to develop obsessions or exacerbate pre-existing ones. Consequently, it is preferable to avoid giving this medicine to someone who is schizo-obsessive if at all feasible [23]. When CBT alone cannot show sufficient progress in a child, medication with an SSRI should be used. Doses should be at the higher end of the suggested range, and the child should be monitored for at least 12 weeks before starting treatment [24].

Considering the limitations of available data and the requirement for drug safety monitoring, antipsychotic augmentation may be an option for individuals not responding to treatment [23]. Selective serotonin reuptake inhibitors (SSRIs) or cognitive behavior therapy combined with exposure and response prevention is advised as first-line treatment [25]. SSRIs are the only medications that are helpful in monotherapy for OCD [4]. Pharmacologic augmentation, thus, with additional medications, is standard in cases where SSRI monotherapy is ineffective, provided there is convincing evidence that adding low-dose neuroleptics to stable SSRIs is beneficial [4]. Although many different agents have been studied in this situation, it is unclear whether they are beneficial. Virtually all of the primary psychiatric diseases are associated with altered cortico-striatal systems and their modulation, which may also assist in explaining common comorbidities of OCD like depression and schizophrenia, which have also been associated with altered processing in these circuits [2]. To distinguish OCD from other diseases, it is crucial to identify the precise cortico-striatal underpinnings of its symptoms, so determining which elements of these circuits' operation result from significant "priors" like heredity or prior experiences like stressor triggers is equally crucial [2].

Treatment-resistant OCD can be managed with various approaches, such as combining an atypical antipsychotic with SSRIs. There is ample documentation of the strong correlation between OCD and attention deficit hyperactivity disorder (ADHD), particularly regarding ADHD in children with OCD. OCD and ADHD frequently have chronic symptoms that last 40-50% of the time [26]. Bipolar disorder (BD) and BD comorbid with OCD patients differed so significantly from one another in terms of psychopathological characteristics (e.g., distinct mood episode onset, history of attempted suicide, seasonality, rapid cycling, and impulsivity) that the comorbidity of BD and OCD may be a separate form of BD, akin to cyclothymic BD [27]. Enhancing parent involvement, utilizing interactive, visual, or multimodal teaching strategies, and incorporating special interests into sessions are some of the ways that empirically supported CBT programs tailored specifically for young people with ASD and anxiety seek to improve child engagement, comprehension, retention, and generalization of CBT skills [18,24]. Diverse treatment strategies and interplay between OCD and other disorders are shown in Table 2.

**Table 2:** Diverse treatment strategies and the interplay between OCD and other disorders OCD: Obsessive-compulsive disorder

Treatment considerations for OCD	Additional insights
Medication dosages	They typically require higher doses than other conditions, with longer reaction times.
Treatment-resistant OCD approaches	We are combining an atypical antipsychotic with a selective serotonin reuptake inhibitor.
Bipolar disorder (BD) and OCD	Significant differences between BD and BD comorbid with OCD patients regarding psychopathological characteristics suggest a separate form of BD akin to cyclothymic BD.

On the other hand, comorbidity does not appear to affect cognitive function; for example, there is no discernible difference between people who experience OCD initially and BD, afterwards, or vice versa [27]. The significant comorbidity of OCD makes it familiar. Pharmacological and psychotherapy interventions are typically used in combination for treatment. Nonetheless, 30% of patients experience severe chronic symptoms with a significant functional impact [28]. Larger areas, such as the dorsolateral prefrontal and posterior regions, may be involved in the pathophysiology of OCD, according to the data from functional magnetic resonance imaging studies. It's also important to keep in mind that OCD is diverse and may include several distinct brain systems that are connected to clinical elements like symptom severity [28]. Recent studies on OCD in neuropsychology and neuroimaging are reviewed in this review. We will also discuss some recent developments in neurological modeling. Discoveries in these domains will update the traditional biology model of OCD. A functional deficiency in the system for context-appropriate dynamic arbitration between model-free and model-based decision-making may cause OCD's rigid dependence on habit [29]. A noteworthy OCD prevalence in schizophrenia is higher than predicted based on computed comorbidity statistics [27].

The hypothesized functional circuits of schizophrenia and OCD have much in common, which could result in co-expression of symptoms [30]. People who suffer from OCD symptoms or schizophrenia symptoms may benefit from antipsychotic drugs to treat these disorders; some psychiatrists may prescribe an antipsychotic and an antidepressant; however, taking these drugs together might have dangerous side effects, including seizures. Instead, other considerations, including the intensity of symptoms, the degree of understanding of the condition, or the signs of co-occurring disorders with the obsessions, should be considered when selecting a drug [30]. Neurotransmitter dysfunction overlaps, but intricate relationships exist, particularly in the dopamine and serotonin systems [4,25]. Compulsions in OCD refer to the recurring behaviors you feel obligated to carry out to alleviate or eliminate your obsessions. These obsessive behaviors are not pleasurable for people with OCD and are not something they want to do. However, they believe their anxiety will worsen if they don't complete them. But compulsions are only helpful in the short term. The compulsions resume shortly after the obsessions reappear. Compulsions take up time and interfere with valuable tasks that you enjoy doing. Put items in a particular order, such as the ones on your dresser. Bathing, cleaning, or repeatedly washing your hands. Neurosurgery is a viable therapeutic option for those with severe symptoms of OCD, along with serotonin reuptake inhibitors and cognitive-behavioral therapy [1]. It may be possible to understand OCD further and enhance clinical results by combining translational neuroscience methods with global mental health.

## Conclusions

In addition to presenting distinct symptoms of compulsions and obsessions, OCD is a neuropsychiatric condition that intersects with many other mental conditions. Due to its chronic nature, it has become one of the most disabling illnesses and is receiving much attention from the international medical community. One of the most important things to remember from the overview is that OCD treatment involves several approaches. Although widely used treatments like SSRIs and CBT have proven successful, some patients still exhibit symptoms resistant to treatment. This has prompted experts to look into the effectiveness of further alternative therapies. Moreover, the broad co-morbidity of OCD with diseases such as BD, schizophrenia, and ADHD emphasizes the complexity of psychiatric disorders and the significance of a thorough diagnosis. The relationship between OCD and BD suggests possible common pathogenic pathways. Other complexity includes the egodystonic aspect of OCD, in which sufferers acknowledge the absurdity of their behavior, the importance of accuracy in diagnostic processes, and help prevent disorders like postpartum psychosis from being mistakenly classified as OCD. Last, but not least, it's important to remember that even while progress has been achieved in diagnosing and treating OCD, some individuals still experience symptoms, emphasizing the need for continued study and the creation of novel treatments.
